# Distinct C_4_ sub‐types and C_3_ bundle sheath isolation in the Paniceae grasses

**DOI:** 10.1002/pld3.373

**Published:** 2021-12-27

**Authors:** Jacob D. Washburn, Josh Strable, Patrick Dickinson, Satya S. Kothapalli, Julia M. Brose, Sarah Covshoff, Gavin C. Conant, Julian M. Hibberd, Joseph Chris Pires

**Affiliations:** ^1^ Plant Genetics Research Unit, USDA‐ARS University of Missouri Columbia MO USA; ^2^ Division of Biological Sciences University of Missouri Columbia MO USA; ^3^ Department of Molecular and Structural Biochemistry North Carolina State University Raleigh NC USA; ^4^ Department of Plant Sciences University of Cambridge Cambridge UK; ^5^ Program in Genetics, Bioinformatics Research Center, Department of Biological Sciences North Carolina State University Raleigh NC USA

**Keywords:** C_4_, C_4_ sub‐types, evolution, photosynthesis

## Abstract

In C_4_ plants, the enzymatic machinery underpinning photosynthesis can vary, with, for example, three distinct C_4_ acid decarboxylases being used to release CO_2_ in the vicinity of RuBisCO. For decades, these decarboxylases have been used to classify C_4_ species into three biochemical sub‐types. However, more recently, the notion that C_4_ species mix and match C_4_ acid decarboxylases has increased in popularity, and as a consequence, the validity of specific biochemical sub‐types has been questioned. Using five species from the grass tribe Paniceae, we show that, although in some species transcripts and enzymes involved in multiple C_4_ acid decarboxylases accumulate, in others, transcript abundance and enzyme activity is almost entirely from one decarboxylase. In addition, the development of a bundle sheath isolation procedure for a close C_3_ species in the Paniceae enables the preliminary exploration of C_4_ sub‐type evolution.

## 1 INTRODUCTION

C_4_ photosynthesis is often considered the most productive mechanism by which plants convert sunlight into chemical energy (Kopriva & Weber, 2021; Niklaus & Kelly, 2019; Sage, 2004; Wang et al., 2012). The C_4_ pathway leads to increased photosynthetic efficiency because high concentrations of CO_2_ are supplied to RuBisCO. Since its discovery in the 1960s (Hatch & Slack, 1966), a unified understanding of the biochemistry underpinning C_4_ photosynthesis has emerged. This basic system comprises a biochemical pump that initially fixes HCO_3_^−^ into C_4_ acids in mesophyll (M) cells. Subsequently, diffusion of these C_4_ acids into a separate compartment, followed by their decarboxylation, generates high concentrations of CO_2_ around RuBisCO. In many C_4_ plants, the release of CO_2_ occurs in bundle sheath (BS) cells (Furbank, 2016; Hatch, 1992; von Caemmerer et al., 2017). Although this pump demands additional ATP inputs, in warm environments where RuBisCO catalyzes high rates of oxygenation (and therefore photorespiration), the C_4_ pathway increases photosynthetic efficiency compared with the ancestral C_3_ state.

Elucidation of the C_4_ pathway was initially based on analysis of sugarcane (*Saccharum* spp. L.) and maize (corn, *Zea mays* L.), which both use the chloroplastic NADP‐DEPENDENT MALIC ENZYME (NADP‐ME) to release CO_2_ in BS cells. However, it became apparent that not all species used this chloroplastic enzyme. For example, *Megathyrsus maximus* (formerly *Panicum maximum*), *Urochloa texanum* (formerly *Panicum texanum*), and *Sporobolus poiretti* used the cytosolic enzyme PHOSPHONENOLPYRUVATE CARBOXYKINASE (PEPCK) (Edwards et al., 1971) to release CO_2_ in the BS, whereas *Atriplex spongiosa* and *Panicum miliaceum* showed high activities of the mitochondrial NAD‐DEPENDENT MALIC ENZYME (NAD‐ME) (Hatch & Kagawa, 1974). These findings led to the consensus that different C_4_ species made preferential use of one C_4_ acid decarboxylase and resulted in the classification of C_4_ plants into one of three distinct biochemical pathways (Edwards et al., 1971; Hatch et al., 1975; Hatch & Kagawa, 1976). According to Furbank (2016), there was some early discussion about whether the sub‐types were mutually exclusive or if one species might employ two or more sub‐types together, but in general, the sub‐types were described as distinct (Hatch, 1987).

For several decades, this description of three sub‐types has been standard practice (Hibberd & Covshoff, 2010; Sheen, 1999) and even used in taxonomic classification (Brown, 1977). However, more recent work has provided evidence that some C_4_ species use multiple C_4_ acid decarboxylases. Maize, for example, was traditionally classified as using NADP‐ME, but evidence has mounted that it and sugarcane both have high activities of PEPCK (Bellasio & Griffiths, 2013; Cacefo et al., 2019; Furbank, 2011; Koteyeva et al., 2015; Majeran et al., 2010; Pick et al., 2011; Sharwood et al., 2014; Walker et al., 1997; Wang et al., 2014; Weissmann et al., 2016; Wingler et al., 1999). This blurring of the NADP‐ME C_4_ sub‐type coincided with observations that many plants with high amounts of PEPCK also contained either NADP‐ME or NAD‐ME (Furbank, 2011). Furthermore, computational models of the C_4_ pathways suggested that BS energy requirements could not be met in a system with only PEPCK decarboxylation (Wang et al., 2014). It has therefore been suggested that PEPCK may never function on its own as a distinct sub‐type (Bräutigam et al., 2014; Furbank, 2011; Wang et al., 2014).

Alternatives to the three sub‐type classification have since been proposed and used in a number of recent publications. These include a two‐sub‐type system (based on the use of NADP‐ME or NAD‐ME), as well as a four‐sub‐type classification placing species into NADP‐ME, NAD‐ME, NADP‐ME + PEPCK, and NAD‐ME + PEPCK sub‐types (Rao & Dixon, 2016; Wang et al., 2014; Washburn et al., 2015). At present, none of these classification schemes have been widely adopted by the community. Moreover, convincing experimental evidence (i.e., transcriptomic or proteomic data) that species traditionally defined as belonging to the PEPCK sub‐type actually use another C_4_ acid decarboxylation enzyme at a higher level than PEPCK is lacking, whereas enzyme activity measurements in the older literature indicate strong PEPCK predominance for several species (Gutierrez et al., 1974; Lin et al., 1993; Prendergast et al., 1987).

**FIGURE 1:**
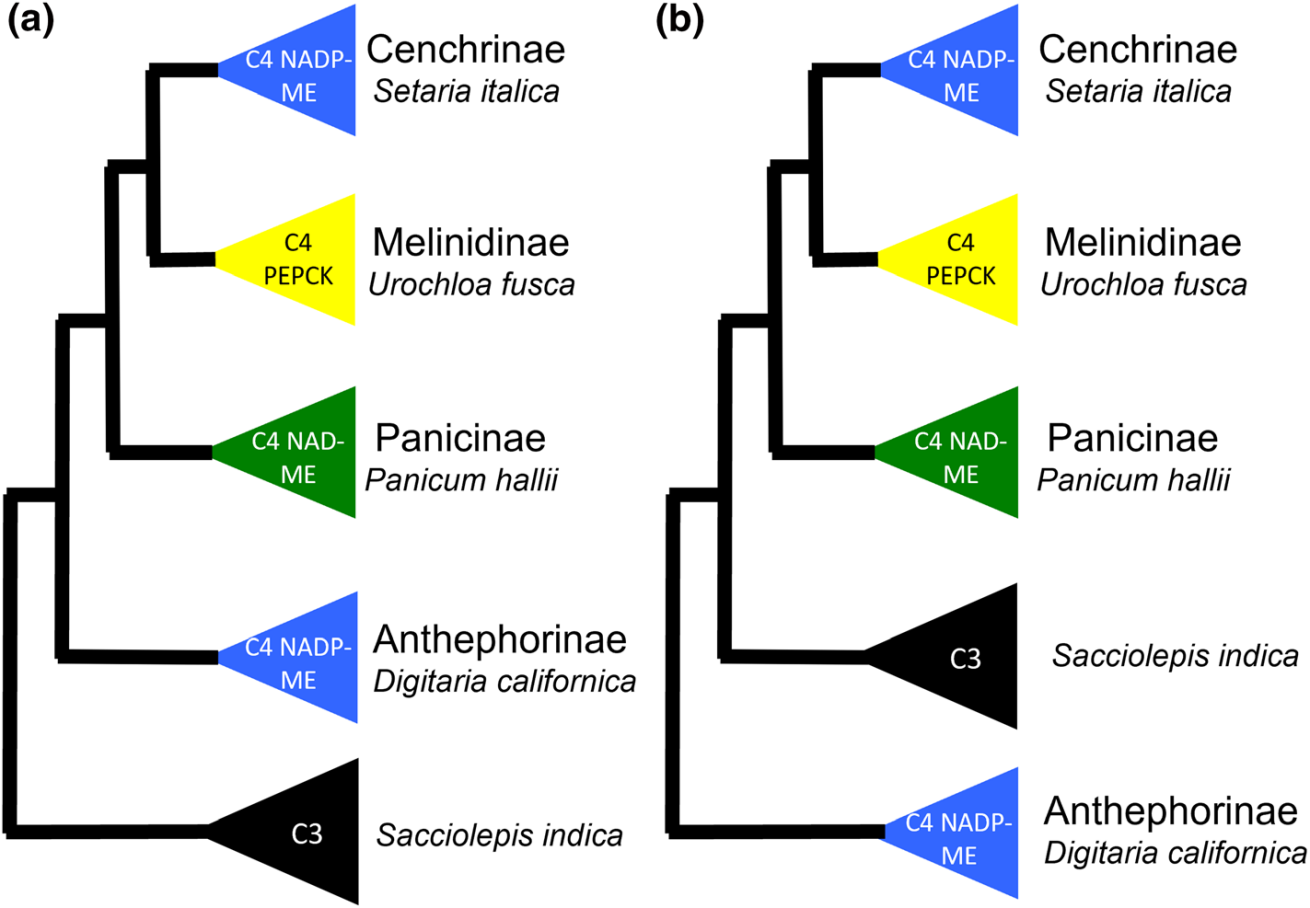
Phylogenetic relationships between a subset of species in the grass tribe Paniceae (Poaceae). The photosynthetic type (C_3_ or C_4_) and C_4_ sub‐type of each species is labeled in the colored triangle next to it. *NAD‐ME*, *NAD‐DEPENDENT MALIC ENZYME*; *NADP‐ME*, *NADP‐DEPENDENT MALIC ENZYME*; *PEPCK*, *PHOSPHONENOLPYRUVATE CARBOXYKINASE*. (a) Phylogeny based on nuclear genes (Vicentini et al., 2008; Washburn et al., 2017). (b) Phylogeny based on chloroplast genes (Washburn et al., 2015)

Only one group of species, the tribe Paniceae (Poaceae) has been documented to contain all three classical biochemical sub‐types of C_4_ photosynthesis together in a pattern consistent with a single C_4_ origin (Sage et al., 2011). The subtribe Cenchrinae consists of species using the classical NADP‐ME C_4_ sub‐type, the subtribe Melinidinae the PEPCK sub‐type, and the Panicinae the NAD‐ME sub‐type (Gutierrez et al., 1974; Lin et al., 1993; Prendergast et al., 1987). The subtribes Cenchrinae, Melinidinae, and Panicineae (CMP) form a well‐supported phylogenetic clade of C_4_ species with many C_3_ species sister to the clade (Grass Phylogeny Working Group II, 2012; Vicentini et al., 2008; Washburn et al., 2015). Studies based solely or predominantly on nuclear genes have confirmed this CMP clade, but nuclear gene phylogenies show a slightly different relationship between the CMP clade and other species in the Paniceae by placing the subtribe Anthephorineae as sister to the CMP clade and the C_3_ relatives then sister to this combined clade. These phylogenies support a CMPA clade of C_4_ species potentially sharing a single C_3_ ancestor (Vicentini et al., 2008; Washburn et al., 2017). This clade is here referred to as the CMP(A) clade in order to indicate the incongruence found between nuclear and chloroplast phylogenies (Figure 1). The analyses here performed are equally valid regardless of the inclusion of Anthephorineae.

How and why C_4_ photosynthesis and its sub‐types evolved has been investigated for many years (Langdale, 2011; Raghavendra, 1980; Rawsthorne, 1992; Sage, 2001, 2004; Sage et al., 2012; Schluter & Weber, 2020). Current hypotheses suggest an intermediate C_3_–C_4_ stage in which a photorespiratory pump operated (Blätke & Bräutigam, 2019; Bräutigam & Gowik, 2016; Heckmann et al., 2013; Mallmann et al., 2014; Sage, 2004; Sage et al., 2012). Each C_4_ sub‐type would require at least some distinct evolutionary innovations, and the question of how or why multiple sub‐types would evolve from the same C_3_ ancestor remains unanswered, although some evidence suggests it could be related to light quality and/or nutrient availability (Arp et al., 2021; Blätke & Bräutigam, 2019; Pinto et al., 2016; Sonawane et al., 2018).

To better determine whether the PEPCK pathway represents a true biochemical sub‐type and investigate the extent to which C_4_ species make use of mixtures of C_4_ acid decarboxylases, global patterns of mRNA abundance were assessed from BS‐ and M‐enriched samples across phylogenetically spaced C_4_ plants that were traditionally defined as exclusively using one of each of the C_4_ sub‐types. These species belong to each subtribe of the CMP(A) described above. The C_3_ species *Sacciolepis indica*, another member of the Paniceae and sister to the CMP(A) clade (sister to CMP in chloroplast phylogeny and sister to CMPA in nuclear phylogeny), was included in the analysis to provide insight into the ancestral state and evolutionary transition from C_3_ to different C_4_ sub‐types (Washburn et al., 2015, 2017). A simple method was developed for isolating BS cells from the C_3_ species *S. indica*.

## 2 MATERIALS AND METHODS

### 2.1 Plant materials

Accessions from five plant species were used in this study: *Setaria italica* yugu1, *Urochloa fusca* LBJWC‐52, *Panicum hallii* FIL2, *Digitaria californica* PI 364670, and *S. indica* PI 338609. More details on the accessions can be found in Washburn et al. (2015) with exception of *P. hallii* FIL2, obtained from Thomas Juenger of the University of Texas at Austin with further details at *P. hallii* v2.0, DOE‐JGI, http://phytozome.jgi.doe.gov/.

Plant materials for RNA sequencing (RNA‐Seq) were grown in controlled growth chambers at the University of Missouri in Columbia. Plants were grown under 16 h of light (from 6:00 a.m. to 8:00 p.m.) and 8 h of darkness with temperatures of 23°C during the day and 20°C at night. Lights were placed between 86 and 88 cm above the plants providing approximately 250 μmol of light at plant level. Plantings were grown in four replicates in a completely randomized design with 32 plants per replicate (except for *S. indica* where plants were smaller and grown with 64 plants per replicate). The third leaf was sampled between 11:00 a.m. and 3:00 p.m. using established leaf rolling and mechanical BS isolation methods with some modifications (see the supporting information) (Chang et al., 2012; Covshoff et al., 2013; John et al., 2014; Sheen & Bogorad, 1985). Due to time and cost constraints, only three of the four replicates (each based on a pool of 32 or 64 plants respectively) were processed for sequencing.

The protocol used for obtaining BS strands in *S. indica* was the same as that used for the C_4_ species. Variations on the amount of time for each blending step were investigated, but only resulted in higher levels of contamination as viewed under a microscope. That said, even when microscopic examination indicated higher contamination levels in some *S. indica* BS samples, transcript abundance levels were qualitatively similar to samples with less apparent contamination. One step that may have been key to the isolation of C_3_ BS strands was the use of leaf rolling on the sampled leaves just prior to the BS strand isolation procedure (Furbank et al., 1985; John et al., 2014). This enables the removal of at least some M sap prior to BS isolation.

M‐enriched samples where not successfully obtained for *S. indica* in this study because of the sensitivity of the C_3_ leaves to rolling. Very small amounts of leaf rolling pressure resulted in the leaves becoming damaged to the point that the purity of M sap obtained from them was called into question. Leaves that were rolled gently enough not to damage the BS strands and contaminate the M sap resulted in sap with insufficient quantities of RNA for sequencing. It is our opinion that M sap could be sampled using (1) low‐input mRNA extraction and sequencing procedures, (2) a more precise instrument for leaf rolling such as that described by Leegood (1985), and/or (3) further experimentation with the developmental stage at which M sap is extracted.

This resulted in a total of 30 samples used for RNA extraction, sequencing, and analysis, consisting of three replicates of BS and M (or whole‐leaf [WL] tissue for the C_3_) respectively for each of the five species.

### 2.2 Sequencing

RNA was extracted using the PureLink® RNA Mini Kit (Invitrogen, Carlsbad, CA, USA), and mRNA‐Seq libraries were constructed and sequenced by the University of Missouri DNA Core Facility using the TruSeq Stranded mRNA Sample Prep Kit (Illumina, Inc., San Diego, CA, USA) and the Illumina HiSeq and NextSeq platforms.

### 2.3 Analysis

Each mRNA sample was quality trimmed and mapped to the *Sorghum bicolor* genome Version 3.1.1 (DOE‐JGI, 2017; Paterson et al., 2009). All species were mapped to *S. bicolor* because reference genomes do not exist for some of the species in this study and the reference genomes that do exist are of variable quality leading to bias. Mapping all species to *S. bicolor*, which is equally related to all, also allows for orthology assignment during the mapping step as opposed to later in the process. This resulted in mapping rates of around 50% for all species. Although this is a low mapping rate compared with typical within species mapping experiments, it is sufficient for comparing the expression of most genes, particularly those that are well conserved across species, such as the photosynthesis‐related genes here examined. Raw sequence was processed using Trimmomatic and Trinity v2.8.4 following the workflows outlined on the Trinity website (Bolger et al., 2014; Grabherr et al., 2011; Haas et al., 2013). This processing included the use of eXpress and Bowtie2 for read mapping and counting as well as edgeR and DESeq2 for differential expression analysis (Langmead & Salzberg, 2012; Li & Dewey, 2011; Love et al., 2014; McCarthy et al., 2012; Robinson et al., 2010). A list of known C_4_ photosynthesis genes was compiled on the basis of the literature; a custom script and BLAST were then used to find the appropriate homologous genes and to compare their relative abundance levels (Bräutigam et al., 2014; Camacho et al., 2009; Chang et al., 2012; Covshoff et al., 2013; John et al., 2014; Rao et al., 2016; Tausta et al., 2014). For comparisons across all cell types and species within this study, the trimmed mean of M‐values (TMM) method described by Robinson and Oshlack (2010) as implemented in DESeq2 was used. Adjusted *p* values were calculated by the software and represent an adjustment to the standard *p* value to account for multiple testing. All scripts and workflows used in the analysis can be found in a Bitbucket repository at https://bitbucket.org/washjake/paniceae_c4_m_bs_mrna/.

### 2.4 Enzyme assays

Enzyme activity assays were performed on the basis of methods described in (Ashton et al., 1990; Marshall et al., 2007). Samples were grown in growth chambers at the University of Cambridge, UK, and growth conditions were matched as closely as possible to those above. The temperature was a constant 20°C, 60% humidity, 300‐μmol light at plant level, and 16 h of light. Plants were sampled at a similar age, growth stage, and leaf to the RNA‐Seq samples. Samples were prepared by grinding leaf tissue with a pestle and mortar in extraction buffer and then centrifuged at 13,000 *g*, and supernatant was taken.

Preliminary experiments were carried out to ensure that all enzyme assays performed linearly across different concentrations. For PEPCK assays, we used the Walker and Leegood method that was developed to reduce proteolysis and which has been used extensively (Häusler et al., 2001; Marshall et al., 2007; Sharwood et al., 2016; Sommer et al., 2012; Walker et al., 1995). PEPCK extraction buffer consisted of 200‐mM bicine–KOH, pH 9.0, 20‐mM MgCl_2_, and 5‐mM DTT. NAD‐ME extraction buffer consisted of 50‐mM HEPES–NaOH pH 7.2, 50‐mM tricine, 2‐mM MnCl_2_, 5‐mM DTT, 0.25% w/v PVP 40000, and 0.5% Triton X‐100. NADP‐ME extraction buffer consisted of 50‐mM HEPES–KOH pH 8.3, 50‐mM tricine, 5‐mM DTT, and 0.1‐mM EDTA.

For PEPCK activity, assay buffer contained of 80‐mM MES–NaOH pH 6.7, 0.35‐mM NADH, 5‐mM DTT, 2‐mM MnCl_2_, 2‐mM ADP, and 50‐mM KHCO_3_. Background rates were measured for 5 min, then 1.2 U of malate dehydrogenase was added, and rates were measured for a further 5 min. Assays were initiated with the addition of 2‐mM phosphoenolpyruvate (PEP). For NAD‐ME activity, assay buffer contained 25‐mM HEPES–NaOH pH 7.2, 5‐mM l‐malic acid, 2‐mM NAD, 5‐mM DTT, and 0.2‐mM EDTA. Background rates were measured for 5 min, and then 24‐mM MnCl_2_ and 0.1‐mM coenzyme A were used to initiate the reaction. For NADP‐ME activity, assay buffer contained 25‐mM tricine–KOH pH 8.3, 5‐mM l‐malic acid, 0.5‐mM NADP, and 0.1‐mM EDTA. Background rates were measured for 5 min, and the assays were initiated with 2‐mM MgCl_2_.

All assays were performed in 96‐well plates at 25°C in a CLARIOstar Plus plate reader (BMG Labtech) in 200‐μl reactions with absorbance at 340 nm measured every 60 s until steady states were reached. Rates were calculated as the rate of reaction from the initial slope of the reaction minus any observed background rate. Rates were normalized to both protein concentration, measured using the Qubit Protein Assay (Life Technologies), and chlorophyll concentration, extracted using 80% acetone and calculated as in Porra et al. (1989).

It should be noted that assaying PEPCK activity is known to be challenging with several potential sources of error. It has been previously reported that the forward (decarboxylation) reaction is about 2.6 times faster than the reverse (carboxylation) reaction (Ashton et al., 1990), but this amount could be greater or less in our species of interest and depending on conditions. Additionally, N‐terminal cleavage and rapid loss of activity have been reported in the assay, although we tried to address this using published methods to reduce proteolysis and loss of activity by extracting with high pH and DTT (Walker et al., 1995, 1997). PEPC activity could potentially contribute to higher than expected levels of PEPCK, but we controlled for this using a high pH buffer (Ashton et al., 1990). Flexibility in the amount of PEPCK produced under different conditions, particularly nitrogen, has also been documented (Arp et al., 2021; Delgado‐Alvarado et al., 2007; Walker et al., 1999). Although care was taken to grow the RNA and enzyme activity samples in similar conditions, they were grown at different institutions and may have experienced some slight differences in environment.

### 2.5 RNA in situ hybridization

Each of the genotypes was grown in a climate‐controlled growth chamber at 50% relative humidity in 16:8 light‐to‐dark cycles at 29.4°C and 23.9°C day and night temperature, respectively. These conditions were different from the original mRNA samples due to the logistics of growth chamber availability. However, because the results appear to support those from the RNA‐Seq at a lower temperature, it does not appear that this temperature difference had a strong impact. Replicates of fully expanded leaf three were harvested from each genotype when plants were at vegetative stage 4 (V4) when the fourth leaf collar was visible. Along the longitudinal length of the leaf blade, midsections of blade tissue were dissected in 3.7% FAA at 4°C. Samples were vacuum infiltrated and fixed overnight at 4°C in 3.7% FAA. Leaf samples were dehydrated through a graded ethanol series (50%, 70%, 85%, 95%, and 100%) with three changes in 100% ethanol; all changes were 1 h each at 4°C except for the last 100% ethanol, which was overnight at 4°C. Samples were then passed through a graded HistoChoice® (Sigma‐Aldrich) series (3:1, 1:1, and 1:3 ethanol:HistoChoice) with three changes in 100% HistoChoice; all changes were 1 h each at room temperature. Samples were then embedded in Paraplast Plus® (McCormick Scientific), sectioned to 10 μm, and hybridized as described previously (Strable & Satterlee, 2021). Two fragments for *PEPCK* consisted of 450 bp of the CDS (synthesized from JSC4‐6 and JSC4‐7 primers) and 456 bp of the 3′ end that included UTR (JSC4‐4 and JSC4‐5). Two fragments for *NADP‐ME* consisted of 790 bp of the CDS (JSC4‐8 and JSC4‐9) and 286 bp of the 3′ end that included UTR (JSC4‐10 and JSC4‐11). Fragments were subcloned into pCR 4‐TOPO (Invitrogen) and confirmed by Sanger sequencing. Antisense or sense strand digoxygenin‐UTP labeled RNA was generated for *PEPCK* and *NADP‐ME* using a DIG RNA labeling kit (Roche). For *PEPCK* hybridizations, equal amounts of the two probes for *PEPCK* were mixed prior to hybridization. Similarly, for *NADP‐ME* hybridizations, equal amounts of the two probes for *NADP‐ME* were mixed prior to hybridization. Primer sequences for RNA in situ probes are provided in Table S4.

## 3 RESULTS

### 3.1 M and BS extraction and distribution of transcripts encoding components of the C_4_ cycle

Four C_4_ species from the Paniceae tribe were chosen to represent the CMP(A) subtribes in the Paniceae (Figure 1). *S. italica* for Cenchrinae (NADP‐ME), *U. fusca* for Melinidinae (PEPCK), *P. hallii for* Panicinae (NAD‐ME), and *D. californica* for Anthephorineae (NADP‐ME). *S. indica* was chosen to represent the closest C_3_ relative to the group.

**FIGURE 2:**
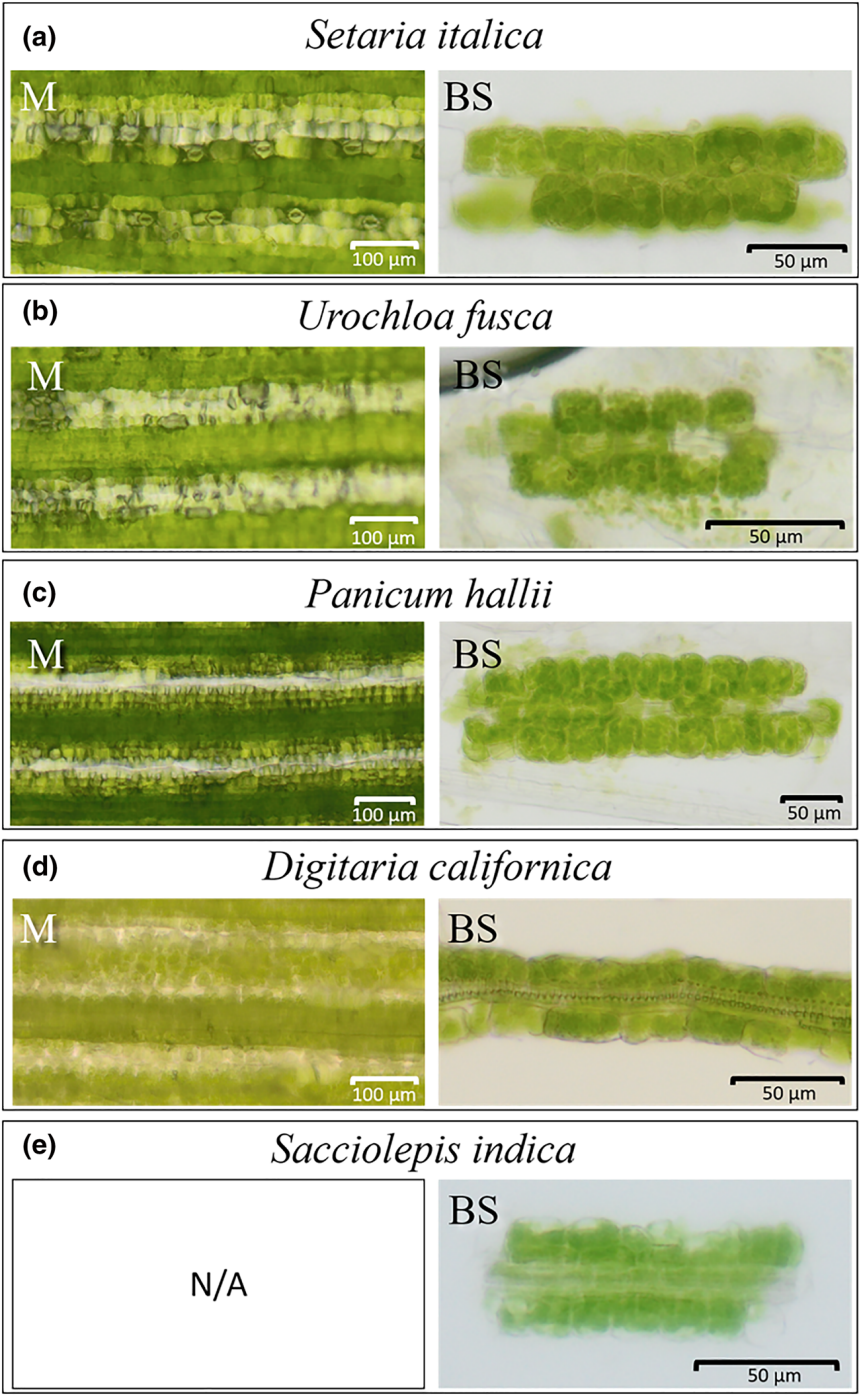
Representative whole‐leaf and bundle strands. Images from leaves that have been rolled to remove mesophyll (M) contents or bundle sheath (BS) strands after isolation. (a) *Setaria italica*, (b) *Urochloa fusca*, (c) *Panicum hallii*, (d) *Digitaria californica*, and (e) *Sacciolepis indica*. All species use the C_4_ pathway except (e), *S. indica*, which is a C_3_ plant. The bands of cells with low chlorophyll content in M images represent the position of mesophyll cells that have collapsed and had their contents expelled during the rolling procedure. Scale bars are depicted

**FIGURE 3:**
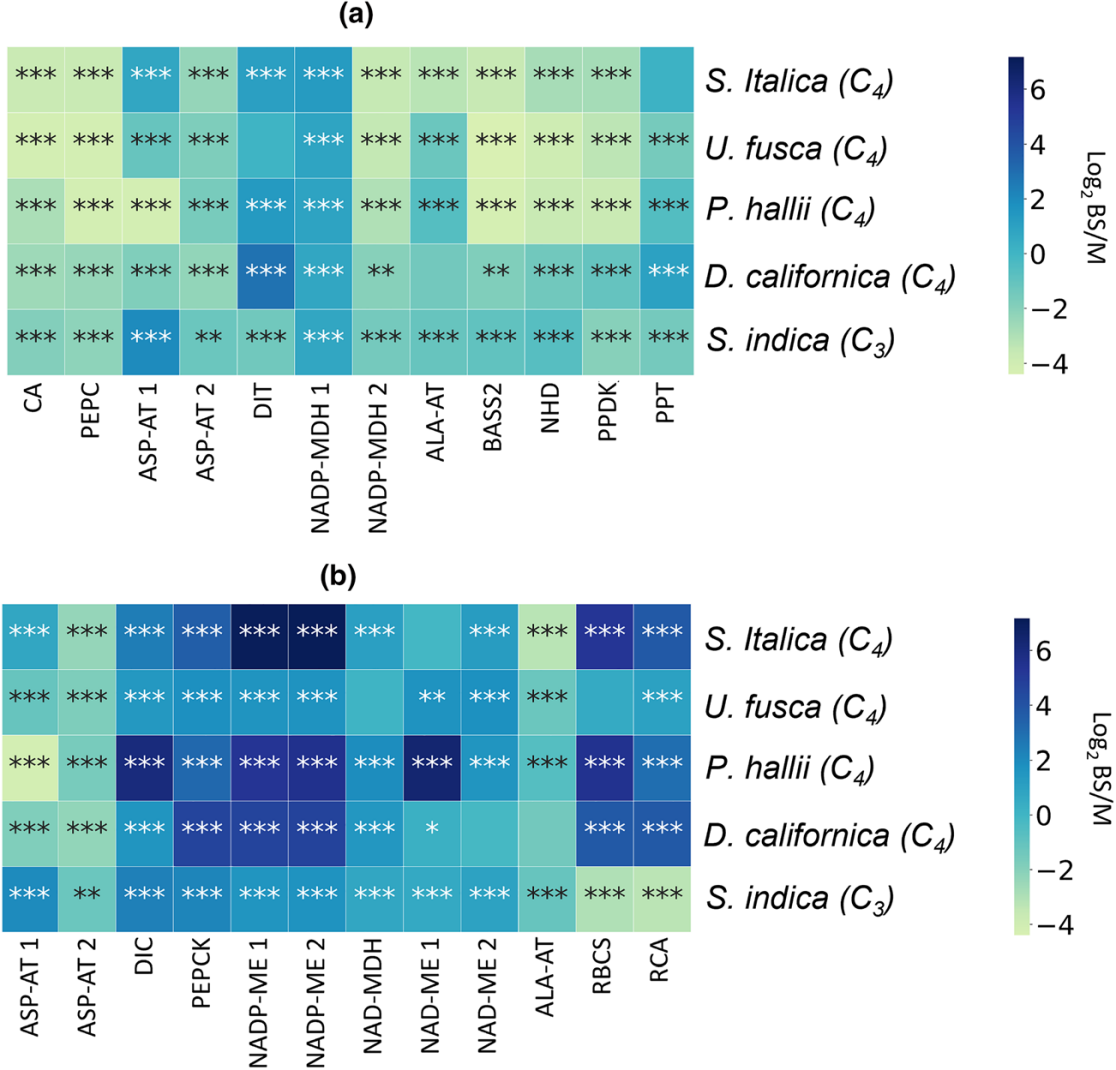
Log_2_ fold change between mesophyll (M)‐enriched and bundle sheath (BS)‐enriched mRNA transcripts. Species used are *Setaria italica*, *Urochloa fusca*, *Panicum hallii*, *Digitaria californica*, and *Sacciolepis indica*. Note that for *S. indica*, a C_3_ species, whole‐leaf data are used in place of M. Genes depicted encode proteins of importance in the C_4_ cycle that are known to be preferentially expressed in either (a) M or (b) BS cells. The number of asterisks in each box represents the *p* value. ^*^ *p* < .05, ^**^ *p* < .01, and ^***^ *p* < .001. *n* = 3. *ALA‐AT*, *ALANINE AMINOTRANSFERASE*; *ASP‐AT*, *ASPARAGINE AMINOTRANSFERASE*; *BASS2*, *SODIUM BILE ACID SYMPORTER 2*; *CA*, *CARBONIC ANHYDRASE*; *DIC*, *MITOCHONDRIAL DICARBOXYLATE CARRIER*; *DIT*, *DICARBOXYLATE TRANSPORTER 1*; *NAD‐MDH*, *NADP‐DEPENDENT MALATE DEHYDROGENASE*; *NAD‐ME*, *NAD‐DEPENDENT MALIC ENZYME*; *NADP‐MDH*, *NADP‐DEPENDENT MALATE DEHYDROGENASE*; *NADP‐ME*, *NADP‐DEPENDENT MALIC ENZYME*; *NHD*, *SODIUM:HYDROGEN ANTIPORTER*; *PEPC*, *PHOSPHOENOLPYRUVATE CARBOXYLASE*; *PEPCK*, *PHOSPHONENOLPYRUVATE CARBOXYKINASE*; *PPDK*, *PYRUVATE*, *ORTHOPHOSPHATE DIKINASE*; *PPT*, *PHOSPHATE/PHOSPHOENOLPYRUVATE TRANSLOCATOR*; *RBCS*, *RUBISCO SMALL SUBUNIT*; *RCA*, *RUBISCO ACTIVASE*. The addition of a space and a number after the enzyme name indicates that multiple genes were mapped that may perform this function

Microscopic examination of leaves of *S. italica*, *U. fusca*, *P. hallii*, and *D. californica*, from which M‐cell contents had been extracted, showed bands of cells containing low chlorophyll content (Figure 2a–d) a phenotype consistent with efficient removal of M content (Covshoff et al., 2013; John et al., 2014). In addition, after mechanical isolation of leaves, BS preparations of high purity for all C_4_ species were generated (Figure 2a–d). Separation of BS strands was also successful for the C_3_ species *S. indica*, something that to our knowledge has not been successful in any other C_3_ species (Figure 2e). Analysis of transcripts derived from important C_4_ genes showed clear differences in abundance between M and BS samples from the C_4_ species. For example, transcripts derived from *CARBONIC ANHYDRASE* (*CA*), *PHOSPHOENOLPYRUVATE CARBOXYLASE* (*PEPC*), and *PYRUVATE*, *ORTHOPHOSPHATE DIKINASE* (*PPDK*) genes preferentially accumulated in M cells (Figure 3a and Table S1). In contrast, transcripts derived from the *RUBISCO SMALL SUBUNIT* (*RBCS*) and *RUBISCO ACTIVASE* (*RCA*) as well as either *NADP‐ME*, *NAD‐ME*, or *PEPCK* were more abundant in BS strands (Figure 3b). The abundance of transcripts relating to C_4_ photosynthesis in the C_3_ species *S. indica* was also consistent with current knowledge of metabolism in the BS of C_3_ species. For example, RBCS and RCA were more abundant in WL samples than in the BS.

### 3.2 Some Paniceae lineages use classical sub‐types, and others mix C_4_ acid decarboxylases

**FIGURE 4:**
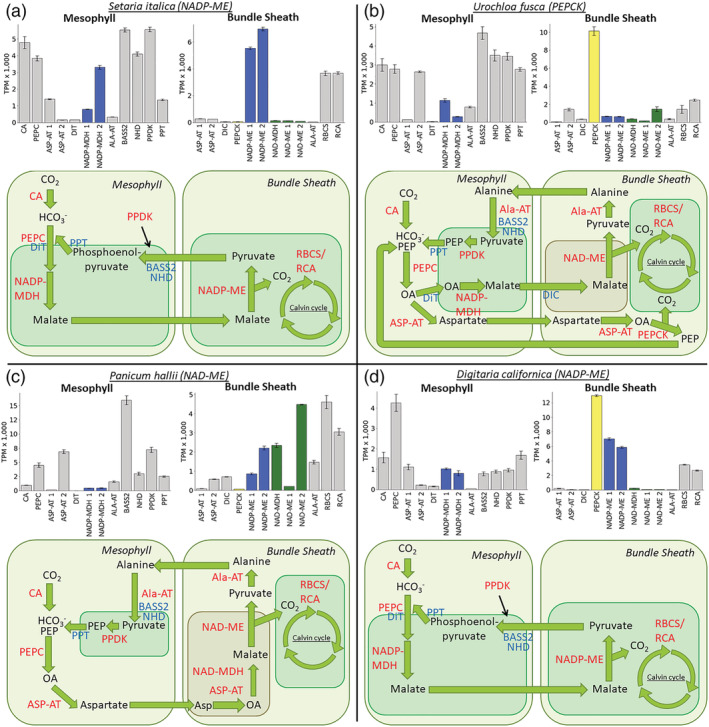
Relative transcript abundance between important C_4_ enzymes within mesophyll (M) and bundle sheath (BS) extracts. Data are displayed for (a) *Setaria italica*, (b) *Urochloa fusca*, (c) *Panicum hallii*, and (d) *Digitaria californica*. The schematics below each histogram indicate the enzyme complement associated with each of the three biochemical sub‐types. *ALA‐AT*, *ALANINE AMINOTRANSFERASE*; *ASP‐AT*, *ASPARAGINE AMINOTRANSFERASE*; *BASS2*, *SODIUM BILE ACID SYMPORTER 2*; *CA*, *CARBONIC ANHYDRASE*; *DIC*, *MITOCHONDRIAL DICARBOXYLATE CARRIER*; *DIT*, *DICARBOXYLATE TRANSPORTER 1*; *NAD‐MDH*, *NADP‐DEPENDENT MALATE DEHYDROGENASE*; *NAD‐ME*, *NAD‐DEPENDENT MALIC ENZYME*; *NADP‐MDH*, *NADP‐DEPENDENT MALATE DEHYDROGENASE*; *NADP‐ME*, *NADP‐DEPENDENT MALIC ENZYME*; *NHD*, *SODIUM:HYDROGEN ANTIPORTER*; *PEPC*, *PHOSPHOENOLPYRUVATE CARBOXYLASE*; *PEPCK*, *PHOSPHONENOLPYRUVATE CARBOXYKINASE*; *PPDK*, *PYRUVATE*, *ORTHOPHOSPHATE DIKINASE*; *PPT*, *PHOSPHATE/PHOSPHOENOLPYRUVATE TRANSLOCATOR*; *RBCS*, *RUBISCO SMALL SUBUNIT*; *RCA*, *RUBISCO ACTIVASE*. The addition of a space and a number after the enzyme name indicates that multiple genes were mapped that may perform this function. Error bars are plus or minus the standard error across replicates (*n* = 3). TPM, transcript per million. Transcript expression is scaled so that the sum of all TPMs is equal to one million

**FIGURE 5:**
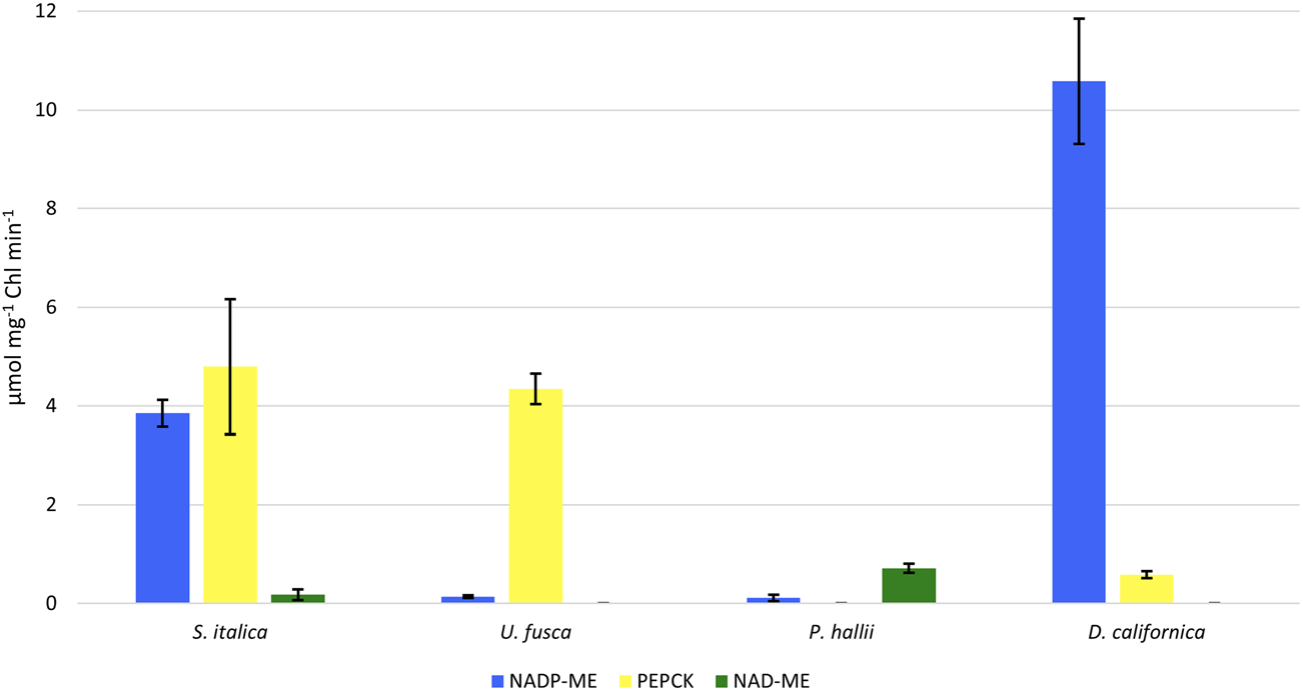
Enzyme activities of C_4_ decarboxylases. *NADP‐DEPENDENT MALIC ENZYME* (*NADP‐ME*), *PHOSPHOENOLPYRUVATE CARBOXYKINASE* (*PEPCK*), and *NAD‐DEPENDENT MALIC ENZYME* (*NAD‐ME*) for *Setaria italica*, *Urochloa fusca*, *Panicum hallii*, and *Digitaria californica*. Error bars represent plus or minus the standard error across replicates (*n* = 3–8)

**FIGURE 6:**
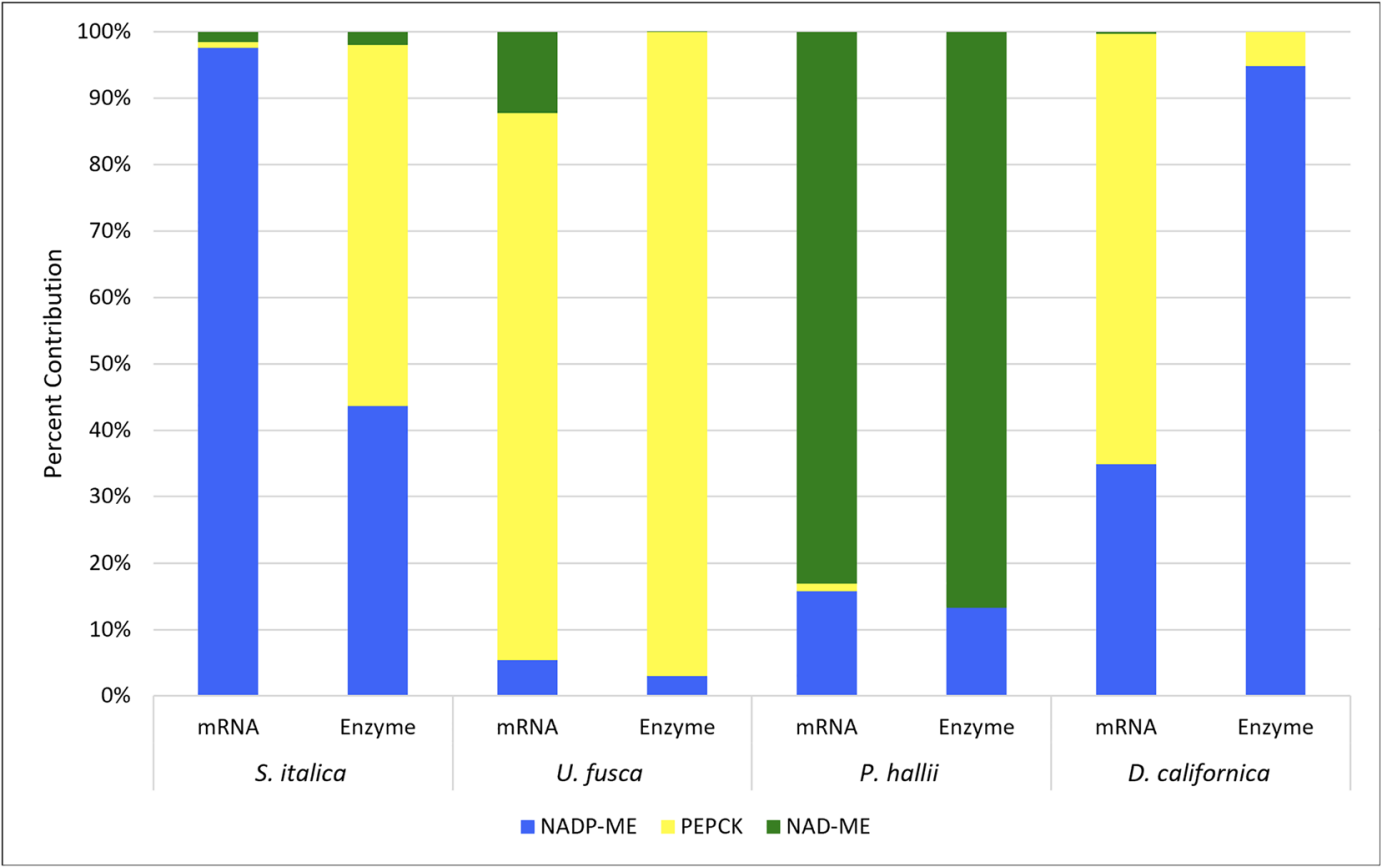
Relative transcript accumulation and enzyme activities of C_4_ decarboxylases. *NADP‐DEPENDENT MALIC ENZYME* (*NADP‐ME*), *PHOSPHOENOLPYRUVATE CARBOXYKINASE* (*PEPCK*), and *NAD‐DEPENDENT MALIC ENZYME* (*NAD‐ME*) transcript accumulation and enzyme activities for *Setaria italica*, *Urochloa fusca*, *Panicum hallii*, and *Digitaria californica*. Values are represented as a percentage of the total of all three decarboxylase values. Enzyme and mRNA data were not collected at the same time but under closely matching environmental conditions (*n* = 3–8)

*S. italica*, classically considered an NADP‐ME sub‐type species, showed high transcript levels for *NADP‐ME* and *NADP‐MDH* in BS and M cells, respectively (Figure 4a). In addition, consistent with the *NADP‐ME* sub‐type, in BS strands of *S. italica*, transcripts encoding *PEPCK*, *NAD‐ME*, *NAD‐MDH*, *ASP‐AT*, and *ALA‐AT* were detected at low levels. Enzyme activity assays also indicated high levels of NADP‐ME in *S. italica* (Figures 5 and 6). Surprisingly, high levels of PEPCK enzyme activity were also found in *S. italica*, but this was not the case for *PEPCK* transcripts. This may be explainable by the differences in growth chamber conditions between RNA samples and enzyme activity samples, differences in cellular locations between the samples (RNA based on BS isolations and enzyme based on WL tissue), contributions of PEPC or other unknown proteins, or well‐established potential sources of error in the PEPCK assay (see Section 2). Previous studies on *S. italica* and many of its close relatives have indicated very low levels of PEPCK consistent with the RNA data here presented (Gutierrez et al., 1974; Lin et al., 1993; Prendergast et al., 1987).

*U. fusca* is classically thought to exclusively use *PEPCK* to release CO_2_ in the BS. The patterns of transcript accumulation in M and BS strands of *U. fusca* are consistent with *PEPCK* functioning in this species with very little to no supplemental decarboxylation from either *NADP‐ME* or *NAD‐ME* (Figure 4b). BS strands contained barely detectable levels of transcripts encoding *NADP‐ME*, low levels for *NAD‐ME*, and very high levels for *PEPCK*. Enzyme activity in *U. fusca* was consistent with transcript abundances, but showed even smaller percentages of NADP‐ME and NAD‐ME relative to PEPCK than the transcriptomes (Figures 5 and 6). In addition, consistent with the cycling of aspartate and alanine between the two cell types, transcripts derived from genes encoding both *ASP‐AT* and *ALA‐AT* were detectable in the two cell types (Figure 4b).

*P. hallii* has been classically considered to use NAD‐ME as its primary decarboxylation enzyme. This assertion is supported by high levels of transcripts encoding *NAD‐ME*, *NAD‐MDH*, *ASP‐AT*, and *ALA‐AT* (Figure 4c). In addition, unexpectedly high levels of transcripts encoding *NADP‐ME* were detected in both the transcripts and enzyme activities of the *P. hallii* BS (Figure 4c). *NAD‐ME* transcript levels were still more than twice those of *NADP‐ME*, and the enzyme assays found much higher relative levels of NAD‐ME than NADP‐ME, indicating a primary use of NAD‐ME (Figures 5 and 6).

In the case of *D. californica*, which is thought to belong to the *NADP‐ME* sub‐type, although transcripts encoding *NADP‐ME* and *NADP‐MDH* were abundant in BS and M samples respectively, *PEPCK* levels were more than double those of *NADP‐ME* in the BS (or perhaps the levels are similar if one considers the possibility that both *NADP‐ME* genes here mapped are resulting in similar functional products). Enzyme assay results showed high levels of NADP‐ME and much lower levels of PEPCK consistent with the traditional sub‐type classification of this species (Figures 5 and 6).

To further confirm or refute these findings, RNA in situ hybridizations were undertaken. Transcript accumulation by in situ hybridization experiments were consistent in signal strength with the RNA‐Seq results from above and indicated that the signals clearly localized to the expected anatomical locations (Figure S1). Previous enzyme assay results for species closely related to the ones sampled here are also extremely consistent with the RNA‐Seq results (Figure S2). It should be noted that the conditions for mRNA, in situ, and enzyme assay sample collection were not identical (see Sections 2 and 4).

### 3.3 C_4_ pathway transporters

Although the decarboxylation enzymes described above have long been used to classify C_4_ sub‐types, transporters are also important to each of the sub‐type pathways (see Figure 4). The transcript abundance of various transporters related to C_4_ photosynthesis was examined. Some transporters had low or undetectable levels such as *DICARBOXYLATE TRANSPORTER 1 (DIT1)*. Others, such *SODIUM BILE ACID SYMPORTER 2 (BASS2)*, *SODIUM:HYDROGEN ANTIPORTER (NHD)*, *MITOCHONDRIAL DICARBOXYLATE CARRIER (DIC)*, and *PHOSPHATE/PHOSPHOENOLPYRUVATE TRANSLOCATOR (PPT)*, had variable levels of transcript abundance across the species and showed no obvious correlation with different C_4_ sub‐types (Figure 4).

### 3.4 C_4_ transcript abundance levels in the closest C_3_ relative to the MPC(A) clade

**FIGURE 7:**
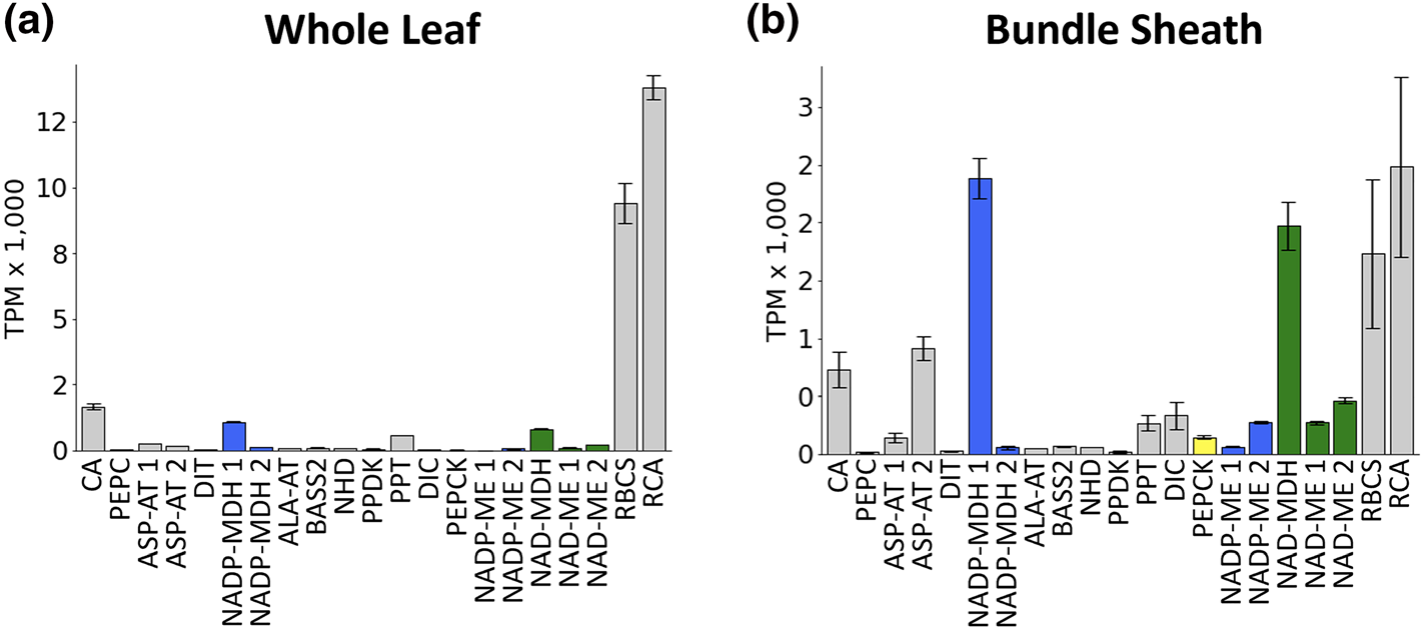
Relative transcript abundance between important C_4_ enzymes within whole‐leaf and bundle sheath (BS) extracts from *Sacciolipis indica*. (a) Whole leaf and (b) bundle sheath. *ALA‐AT*, *ALANINE AMINOTRANSFERASE*; *ASP‐AT*, *ASPARAGINE AMINOTRANSFERASE*; *BASS2*, *SODIUM BILE ACID SYMPORTER 2*; *CA*, *CARBONIC ANHYDRASE*; *DIC*, *MITOCHONDRIAL DICARBOXYLATE CARRIER*; *DIT*, *DICARBOXYLATE TRANSPORTER 1*; *NAD‐MDH*, *NADP‐DEPENDENT MALATE DEHYDROGENASE*; *NAD‐ME*, *NAD‐DEPENDENT MALIC ENZYME*; *NADP‐MDH*, *NADP‐DEPENDENT MALATE DEHYDROGENASE*; *NADP‐ME*, *NADP‐DEPENDENT MALIC ENZYME*; *NHD*, *SODIUM:HYDROGEN ANTIPORTER*; *PEPC*, *PHOSPHOENOLPYRUVATE CARBOXYLASE*; *PEPCK*, *PHOSPHONENOLPYRUVATE CARBOXYKINASE*; *PPDK*, *PYRUVATE*, *ORTHOPHOSPHATE DIKINASE*; *PPT*, *PHOSPHATE/PHOSPHOENOLPYRUVATE TRANSLOCATOR*; *RBCS*, *RUBISCO SMALL SUBUNIT*; *RCA*, *RUBISCO ACTIVASE*. The addition of a space and a number after the enzyme name indicates that multiple genes were mapped that may perform this function. Error bars are plus or minus the standard error across replicates (*n* = 3)

Transcript abundance levels from *S. indica* (C_3_) were generally consistent with expectations for a C_3_ species (Figures 3 and 7). RBCS and RCA were more highly expressed in WL tissue than in BS extracts. Transcripts related to C_4_ photosynthesis were also expressed at a low level in both WL and BS.

Comparisons between the *S. indica* (C_3_) BS‐enriched and WL samples showed significant (adjusted *p* value <.001) BS overabundance for 912 gene models and WL overabundance for 746 gene models (Table S2). Of these overabundant genes, significant Gene Ontology (GO) enrichment (adjusted *p* value <.05) was found for 16 different GO terms for the BS and 14 GO terms for the M (Table S2). The different GO terms enriched in BS cells related to diverse processes including cellular transport, molecular binding, and cell wall and membrane components. The WL GO terms related to photosystems I and II, photosynthesis, and other processes (Table S2).

### 3.5 Gene expression in C_3_ versus C_4_ BS cells

The experimental design provides the opportunity to compare the BS expression from a C_3_ species against BS expression in four closely related C_4_ species. A total of 357 gene models displayed significantly (*p*_adj_ < .001, log_2_ fold change >2) higher transcript abundance levels over the C_3_ BS in all four C_4_ species in the analysis (Table S3). Many of these genes are expected, such as photosystem I and II subunits, cytochrome b_6_f, cyclic electron chain proteins, Calvin cycle proteins, cellulose synthase, *Pectinacetylesterase*, starch synthase, and others. The remaining genes are potential candidates involved in C_4_ photosynthesis that deserve further molecular and biochemical investigation (Table S3).

## 4 DISCUSSION

### 4.1 The PEPCK sub‐type

The dominance of *PEPCK* transcript and enzyme activity over *NADP‐ME* and *NAD‐ME* in *U. fusca* provides evidence for the biological relevance of the classical PEPCK sub‐type (Figures 4, 5, 6 and S2). These data contrast with proposals that PEPCK cannot function on its own but rather is always ancillary to one of the other two C_4_ acid decarboxylases (Bräutigam et al., 2014; Furbank, 2011; Wang et al., 2014). Although this notion may be true in some cases, the current results suggest that it is not the case for *U. fusca*. Furthermore, our findings are supported by earlier measurements of enzyme activity made within the subtribe Melinidinae, where PEPCK was also shown to be highly dominant over the other sub‐types (Gutierrez et al., 1974; Lin et al., 1993; Prendergast et al., 1987), and also indicate that these differences in activity are due to differences in steady‐state transcript abundance rather than posttranscriptional modifications that act to suppress accumulation of NADP‐ME and NAD‐ME.

### 4.2 C_4_ sub‐type mixing

Of the four C_4_ species examined, *P. hallii* and *D. californica* show the most evidence of sub‐type mixing. The potential for mixing has previously been considered in *Panicum virgatum*, a close relative of *P. hallii* (Meyer et al., 2014; Rao et al., 2016; Rao & Dixon, 2016; Zhang et al., 2013). Rao et al. (2016) suggested that the high abundance of *NADP‐ME* transcripts may be accounted for by posttranscriptional or translational modifications, but experimental evidence for testing that hypothesis is lacking.

*D. californica* also showed some evidence of sub‐type mixing. In this case, *NADP‐ME* and *PEPCK* transcripts were both reasonably abundant. Although this species is classically considered to belong to the NADP‐ME sub‐type, transcripts encoding *PEPCK* were more than double the abundance of those of *NADP‐ME*. *ASP‐AT* transcript levels, which are also associated with the *PEPCK* sub‐type, were high as well. However, enzyme activity data do not support this idea with NADP‐ME having much higher levels than PEPCK. Some of these differences between transcript and enzyme levels could be due the enzyme activity assays being carried out on samples from a different growth chamber with somewhat different conditions than the RNA‐Seq samples (see Section 2).

### 4.3 *S. indica* and the C_3_ BS

Analysis of transcript abundance in M and BS cells from C_3_ species that are closely related to C_4_ species is critical to understanding how C_4_ photosynthesis evolved and how it can be engineered for enhanced crop production. Although transcripts loaded onto ribosomes in the BS of C_3_ *Arabidopsis thaliana* have been assessed, and this analysis provided insight into the role of the BS in eudicot plants (Aubry et al., 2014), to our knowledge, there are no equivalent data from a monocotyledonous lineage in which both C_3_ and C_4_ species are found. The ability to mechanically isolate intact BS cells indicates that *S. indica* has very strong BS cell walls, similar to those found in C_4_ species. However, all other currently available data including phylogenetic placement and RNA‐Seq from this study are consistent with *S. indica* being a C_3_ species. The relatively high levels of C_4_ related transcripts in the BS of *S. indica* are consistent with previous work on cells around the veins of C_3_ plants (Brown et al., 2010; Hibberd & Quick, 2002; Shen et al., 2016). Together, these data support the concept that some C_3_ species are preadapted to adopt the C_4_ mechanism (Brown et al., 2011; Burgess et al., 2019; Christin et al., 2009, 2015; Gould, 1989; Reyna‐Llorens et al., 2018; Washburn et al., 2016; Williams et al., 2016). Another potential interpretation of these data is that perhaps *S. indica* represents a step on the pathway to becoming a C_3_–C_4_ intermediate, or a reversion to C_3_ photosynthesis from an ancestral C_3_–C_4_ intermediate (Bräutigam & Gowik, 2016; Sage, 2004).

### 4.4 The *S. indica* C_3_ BS shows marked similarities and differences to the BS in other species

Aubry et al. (2014) investigated the functions of *A. thaliana* BS cells by labeling ribosomes within the cell type and sequencing the mRNA resident in the ribosomes. In general, our *S. indica* BS cells displayed similar patterns to those seen in *Arabidopsis*. Of the 912 significantly overabundant gene models in the *S. indica* BS, 50 of them have *Arabidopsis* homologs that were significantly overabundant in BS cells within the Aubry study (Aubry et al., 2014). These genes have annotated functions relating to transport (nucleotide, peptide, amino acid, sulfate, metals, and ABS transporters), metal handling, transcription regulation, protein degradation, cell wall modification, amino acid metabolism, hormone metabolism, and ATP synthesis. Other functional annotations present in both the *Arabidopsis*‐ and *S. indica*‐upregulated BS gene sets (but not necessarily from homologous genes) included nitrogen metabolism, glutamine synthetase, tryptophan, ethylene‐induced signaling and regulation, lipid metabolism, trehalose metabolism, phenylpropanoid metabolism, and sulfur regulation (Table S2).

Similarly to *Arabidopsis* and maize, several trehalose metabolism genes were found to be overexpressed within the *S. indica* BS, supporting the hypothesis that metabolism of trehalose is an ancestral BS function (Aubry et al., 2014; Chang et al., 2012). The data are also consistent with the hypothesis that BS cells play an important role in sulfur transport and metabolism and in nitrogen metabolism (Aubry et al., 2014). We do note, however, that some sulfur metabolism‐related genes shown to be enriched in *Arabidopsis* BS were actually found to be depleted in the *S. indica* BS. Overall, the *S. indica* BS samples are highly consistent with previous studies on *Arabidopsis* and rice BS, indicating both the validity of the mechanical C_3_ BS isolation done here and the conservation of C_3_ functions across these divergent species (Aubry et al., 2014; Hua et al., 2021).

### 4.5 The evolution of three C_4_ sub‐types in the MPC(A)

For the majority of C_4_ genes examined, all five species appear to use orthologous genes, or at least their transcripts mapped to the same *S. bicolor* gene (Figures 4 and 7). This is based on the common assumption that the highest expressed gene in each species/tissue is the one being used. For CA, PEPC, PPDK, PPT, NHD, ALA‐AT, NAD‐MDH, PEPCK, and NAD‐ME, the highest expressed gene in all species was clearly the same, although in some species, the gene expression was so low that it is likely nonfunctional. NADP‐MDH, NADP‐ME, and ASP‐AT are less clear with the highest gene being different between some species but also often having high abundance levels for both genes, making it hard to conclude that the lower gene is not relevant. The lack of good genomic resources for all species involved makes it difficult to conclude if the same genes are in fact being used by all species, and therefore potentially the result of a single recruitment, or if the genes are simply close homologs and recruited to C_4_ separately in different lineages.

Ancestral state reconstructions were also performed on the basis of the transcript abundance and enzyme activity data; however, these analyses were inconclusive and have been excluded due to the low phylogenetic sampling of the clade within this study.

## 5 CONCLUSIONS

We found that at least one species in the tribe Paniceae appears to use PEPCK decarboxylation exclusively or nearly so, whereas the other species examined appear to be of mix sub‐type. Analysis of the C_3_ species *S. indica*, based on the BS isolation method here developed, showed low levels of C_4_ transcripts and an amenability to mechanical BS separation procedures not previously seen in C_3_ species. These observations lead us to hypothesize that *S. indica* may lie somewhere on the spectrum of C_3_–C_4_ intermediates or represent a reversion from an ancestral C_3_–C_4_ intermediate.

## CONFLICT OF INTERESTS

The Authors did not report any conflict of interest.

## AUTHOR CONTRIBUTIONS

All authors contributed to drafting and revising the manuscript. J. D. W., J. C. P., G. C. C., S. C., and J. M. H. conceived the work and experimental design. J. D. W., S. C., S. S. K., and J. M. B. developed and performed the leaf rolling experiments. J. D. W. performed the bioinformatic analysis. J. S. performed the RNA in situ hybridization experiments. P. D. and J. M. H. designed and performed the enzyme assay experiments. J. D. W. has agreed to serve as the author responsible for contact and communication.

## Supporting information


**Table S1.** Differentially expressed genes between M (or whole leaf for *S. indica*) and BS cells of all species here examined. Same data as displayed in Figure 3.

**Table S2.** Differentially expressed genes between the whole leaf and bundle sheath of *Sacciolepis indica*.

**Table S3.** Differentially expressed genes that are upregulated in all four C4 species bundle sheath cells and down regulated in *Sacciolepis indica* bundle sheath.

**Table S4.** Primer sequences used for in situ hybridization experiments.

**Data S1.** Supporting Information

**Figure S1.** PHOSPHOENOLPYRUVATE *CARBOXYKINASE* (*PEPCK*) and *NADP‐DEPENDENT MALIC ENZYME* (*NADP‐ME*) transcript accumulation in fully expanded leaves. Transverse sections through midpoints of fully expanded leaf 4 hybridized with antisense and sense RNA. Toluidine blue‐O staining of fully expanded leaves. ad, adaxial; ab, abaxial. Scale bars, 200 μm.
**Figure S2.** PCK, NADP‐ME, and NAD‐ME enzyme activity levels in different Paniceae species. Enzyme activity levels for NAPD‐ME, PEPCK (PCK), and NAD‐ME displayed as a percentage of the sum of the activity of all three enzymes for: a) species in the subtribe Cenchrinae, b) species in the sub‐tribe Melinidinae, and c) species in the sub‐tribe Panicinae. Data taken from Gutierrez et al., 1974. Planta 119:279–300, Prendergast et al., 1987. Funct. Plant Biol. 14:403–420, Lin et al., 1993. Funct. Plant Biol. 20:757–769

## Data Availability

Sequence data are available on NCBI SRA (https://www.ncbi.nlm.nih.gov/sra) under Accession Number PRJNA475365.
