# Supplementary material for: Distinct C4 sub‐types and C3 bundle sheath isolation in the Paniceae grasses
Source: Plant Direct. 2021 Dec 27;5(12):e373. doi: 10.1002/pld3.373 (PMC8711749; doi:10.1002/pld3.373)
Supplement: Supplementary file 5 — Data S1. Supporting Information [file PLD3-5-e373-s004.docx]

**Supplemental Protocol 1: Leaf Rolling and Bundle Sheath Protocol**

Modified by Jacob Washburn from Sheen (1995), Covshoff et al. (2013), and John et al. (2014)

Preparation

1) Treat glass plate with 0.1 N NaOH for >30 min and rinse with deionized water. **Or** Clean plate with soap and water, then clean well with RNASE away.

2) Fit glass plate on a box of ice and allow to cool before beginning procedure.

3) Put 500µl of Lysis Buffer from the PureLink RNA mini kit into a 2ml round bottom tube and place on ice.

4) Place 50ml of Bundle Sheath Buffer 1 into a 50ml tube and place on ice.

5) Wash rolling device (25 ml pipette or wall paper roller) and glass plate with RNASE away.

6) Be sure that both the roller and the glass are dry of RNASE away before proceeding. Glass may collect moisture from the air which is ok.

Extraction

7) Harvest one leaf just above the ligule using a razor blade

8) Remove the leaf’s midrib using razor.

9) Cut the leaf into ~3-5 cm strips using razor. Lay strips flat onto glass plate and line them up to make the rolling process efficient.

10) Quickly roll the first strip and collect the liquid (sap) released using a pipette (liquid may be on glass plate and/or roller). Immediately place the liquid in the lysis buffer tube and pipette up and down a few times to mix well. Note: sometimes it is helpful to pipette 5ul of lysis buffer onto the spot where the leaf liquid is in order to more easily capture it.

11) Cut rolled leaf strip into ~2mm by 2mm squares and place in Bundle Sheath buffer 1.

12) Repeat steps 10 and 11 for all remaining leaf strips on the glass plate.

13) Repeat steps 5-12 with all remaining leaf samples. Note: it is important to periodically check a rolled leaf strip under the microscope to insure that BS cells are remaining intact through the rolling process.

Sample Processing

14) Leaf rolling extract, mixed with Lysis Buffer can be left on ice for a few hours while the BS extraction process is carried out. Once BS cells are placed in -80 for storage, proceed immediately to the remainder of the RNA extraction procedure for leaf rolled extract.

15) Place BS buffer 1 with the contained leaf pieces into a blender.

16) Pulse on low for 10 seconds. When the spin comes down pulse again for 10 seconds (for a total of 3 pulses)

17) Filter through a 60 µM mesh (Millipore). Use a small metal spatula to gently stir the leaf bits off the mesh to facilitate drainage. (To make the filter apparatus, cut a 50 ml Falcon tibe in half and cut out the center of the cap. Place the mesh in the cap and screw onto the tube.)

18) Pour about 80-100ml of BS Buffer 2 back through the net to return the leaf debris to the blender. Make sure to add enough buffer to just cover the blender blades.

19) Blend at high speed for one minute.

20) Filter through mesh as in step 5, eluting into beta-mercaptoethanol waste.

21) Repeat steps 18-20 two more times

22) After the final filtration, unscrew the filter from the tube and lay the filter (with BS cells) on a few paper towels to wick away excess moisture. Wrap in a foil packet and drop into liquid nitrogen. Store in -80 freezer until ready to extract RNA using a standard mortar and pistil method.

**References**

**Covshoff S, Furbank RT, Leegood RC, Hibberd JM** (2013) Leaf rolling allows quantification of mRNA abundance in mesophyll cells of sorghum. Journal of Experimental Botany **64:** 807-813

**John CR, Smith-Unna RD, Woodfield H, Covshoff S, Hibberd JM** (2014) Evolutionary Convergence of Cell-Specific Gene Expression in Independent Lineages of C4 Grasses. Plant Physiology **165:** 62-75

**Sheen J** (1995) Methods for mesophyll and bundle sheath cell separation. *In* DW Galbraith, HJ Bohnert, DP Bourque, eds, Methods in Plant Cell Biology, Vol 49. Academic Press, Orlando, pp 305-314
