# Supplementary material for: Distinct C4 sub‐types and C3 bundle sheath isolation in the Paniceae grasses
Source: Plant Direct. 2021 Dec 27;5(12):e373. doi: 10.1002/pld3.373 (PMC8711749; doi:10.1002/pld3.373)
Supplement: Supplementary file 6 — Figure S1. PHOSPHOENOLPYRUVATE CARBOXYKINASE (PEPCK) and NADP‐DEPENDENT MALIC ENZYME (NADP‐ME) transcript accumulation in fully expanded leaves. Transverse sections through midpoints of fully expanded leaf 4 hybridized with antisense and sense RNA. Toluidine blue‐O staining of fully expanded leaves. ad, adaxial; ab, abaxial. Scale bars, 200 μm. Figure S2. PCK, NADP‐ME, and NAD‐ME enzyme activity levels in different Paniceae species. Enzyme activity levels for NAPD‐ME, PEPCK (PCK), and NAD‐ME displayed as a percentage of the sum of the activity of all three enzymes for: a) species in the subtribe Cenchrinae, b) species in the sub‐tribe Melinidinae, and c) species in the sub‐tribe Panicinae. Data taken from Gutierrez et al., 1974. Planta 119:279–300, Prendergast et al., 1987. Funct. Plant Biol. 14:403–420, Lin et al., 1993. Funct. Plant Biol. 20:757–769 [file PLD3-5-e373-s006.pdf]

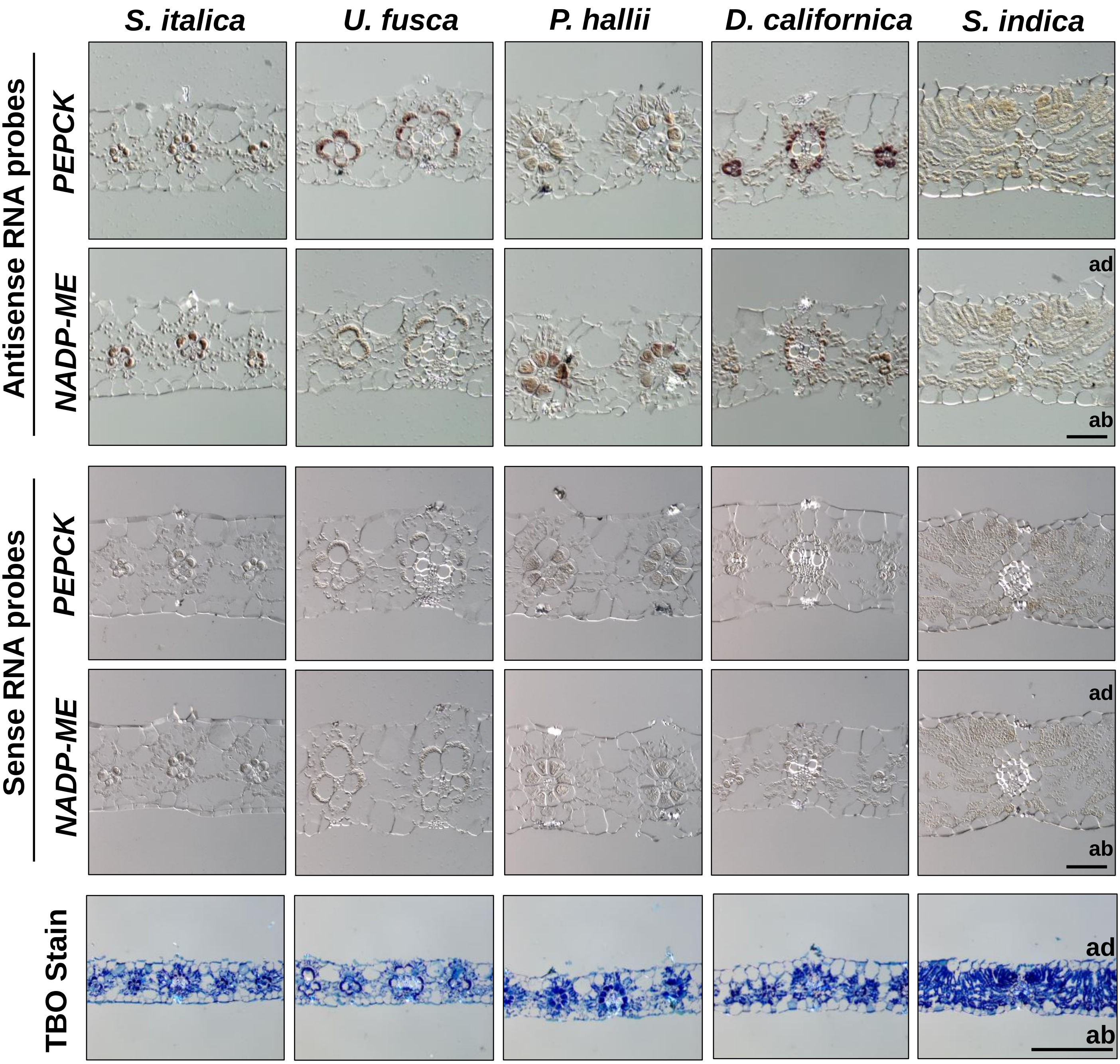

Supplemental Figure 1. PHOSPHOENOLPYRUVATE *CARBOXYKINASE* (*PEPCK)* and *NADP-DEPENDENT MALIC ENZYME* (*NADP-ME*) transcript accumulation in fully expanded leaves. Transverse sections through midpoints of fully expanded leaf 4 hybridized with antisense and sense RNA. Toluidine blue-O staining of fully expanded leaves. ad, adaxial; ab, abaxial. Scale bars, 200 µm.

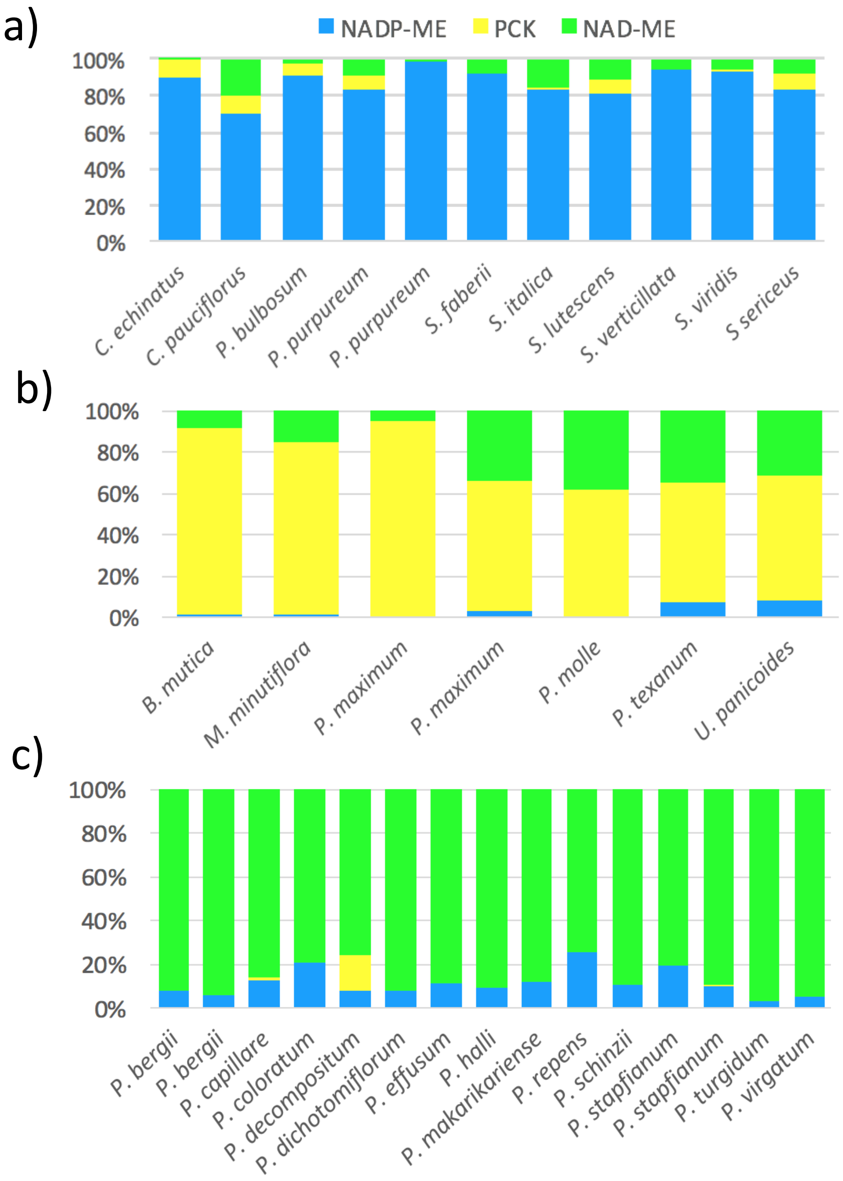

Supplemental Figure 2. PCK, NADP-ME, and NAD-ME enzyme activity levels in different Paniceae species.

Enzyme activity levels for NAPD-ME, PEPCK (PCK), and NAD-ME displayed as a percentage of the sum of the activity of all three enzymes for: a) species in the subtribe Cenchrinae, b) species in the sub-tribe Melinidinae, and c) species in the sub-tribe Panicinae. Data taken from Gutierrez et al., 1974. Planta 119:279-300, Prendergast et al., 1987. Funct. Plant Biol. 14:403-420, Lin et al., 1993. Funct. Plant Biol. 20:757-769.

Supplemental Table 4. Primer sequences for *in situ* hybridization.					
Primer ID	SEQUENCE	GENE	GENE ID	Probe size	Location
JSC4-4	GAGAATCTCCTGAGGCTCGG	GRMZM2G001696	PCK	456	3' end
JSC4-5	ACAGGGGGCAAGATACAAGC	GRMZM2G001696	PCK		3' end
JSC4-6	AGATGGTCATCATGGGCACG	GRMZM2G001696	PCK	450	CDS
JSC4-7	ATGGTATCTTGGCGTTGGGG	GRMZM2G001696	PCK		CDS
JSC4-8	AGGGATGGACACTACTTGCG	GRMZM2G085019	NADP-ME	790	CDS
JSC4-9	AATACGTCTGCTCTGCCAGG	GRMZM2G085019	NADP-ME		CDS
JSC4-10	AGAACTGCATGTACACTCCCG	GRMZM2G085019	NADP-ME	286	3' end
JSC4-11	GAGATCTGACTCGTCCAGCC	GRMZM2G085019	NADP-ME		3' end
